# Conventional gene stacking as a strategy to improve chickpea resistance to Ascochyta blight

**DOI:** 10.3389/fpls.2025.1733694

**Published:** 2026-01-28

**Authors:** Basel Alaskar, Fateh Khatib, Antonious Al-Daoude

**Affiliations:** 1 Department of Plant Protection, Aleppo University, Aleppo, Syria; 2 Department of Molecular Biology and Biotechnology, Atomic Energy Commission of Syria (AECS), Damascus, Syria

**Keywords:** chickpea, chitinase, detached-leaf assays, resistance breeding, vst-1

## Abstract

The main disease that affects chickpea production worldwide is Aschochyta blight (AB), caused by the fungus *Aschochyta rabiei*. The identification of cultivars with stacking resistance genes is crucial for controlling these diseases. This work aimed to evaluate the effect of stacking two resistance-related genes, chitinase and vst-1, on disease response in chickpea (*Cicer arietinum* L.). Gene stacking was achieved through conventional hybridization between three transgenic inbred lines: N292 and N346 (both carrying chitinase), and N52 (carrying vst-1). PCR confirmed the stable inheritance of both transgenes in F1 and F2 generations, although segregation ratios deviated from Mendelian expectations. Functional assays were conducted using protein extracts to test inhibition of fungal spore germination and mycelium formation, followed by detached-leaf and whole-plant infection assays. Protein extracts from stacked lines significantly reduced spore germination (up to 90% inhibition, P < 0.01) and suppressed mycelium development compared to controls. Detached-leaf assays revealed a reduced disease severity in stacked lines (mean DS = 74 vs. 89 in controls), while whole-plant assays confirmed lower severity scores (mean 4–6 vs. 8 in controls) despite no reduction in infection incidence. The hybrid N346 × N52 exhibited the strongest resistance phenotype across assays. These results demonstrate that stacking chitinase and vst-1 increases tolerance to *A. rabiei* in chickpea by reducing disease severity, providing a promising strategy for developing tolerant cultivars. This study is a successful tool for developing gene stacking technology in crops to contribute to improving the resistance of chickpea plants to Ascochyta disease.

## Introduction

Ascochyta blight is a major pathogen of chickpea. This fungus infects all aerial parts of the plant and causes severe losses that may reach 100% of the crop under favorable environmental conditions (Fanning et al., 2022; Pastor et al., 2022). This pathogen has been recorded in more than 40 chickpea-producing countries, causing severe economic losses in the global market (Manjunatha et al., 2018). High humidity and cloudy weather for a long period of time contribute to the rapid development of the pathogen’s symptoms and its rapid spread in chickpea fields (Getaneh et al., 2021). AB epidemics are from infected stubble or seed. Infected crop stubble probably represents the largest reservoir of primary inoculum in chickpea-growing regions (Crociara et al., 2022). Currently, AB is a very challenging disease to manage (Singh et al., 2022), and there is a heavy reliance on fungicides for within-season control of the disease. Genetic resistance is quantitative in nature, and even the most resistant cultivars only partially inhibit pathogen damage (Gayacharan et al., 2020; Sharma and Ghosh, 2016). Rapid evolution of the pathogen threatens the future efficacy of both new sources of resistance and chemical control tools (Bar et al., 2021; Sharma and Ghosh, 2016). A comprehensive understanding of the pathogen’s environment and biology has enabled scientists to identify new sources of resistance and reduce pathogen pressure. Developing Ascochyta-resistant cultivars remains one of the most effective, environmentally safe, and economically viable strategies (Crutcher et al., 2022), This is limited by limited genetic diversity, linkage barriers, and the rapid collapse of resistance due to pathogen evolution (Gao, 2018; Yang et al., 2024). It must be noted that the main reason for the ineffectiveness of disease resistance genes in recent years is the result of the dynamic evolution of these pathogens and their ability to produce new pathogenic strains, which leads to the breakdown of the resistance trait and consequently huge losses in crops. (Yang et al., 2024). Genetic engineering is a powerful and effective tool for overcoming numerous obstacles. Its application has revolutionized plant biology and biotechnology through precise and targeted genome modifications, providing new approaches to genetically improve plant disease resistance and accelerate the breeding of resistant crops (Yin and Qiu, 2019). Genetic engineering offers an alternative, enabling the introduction of resistance genes across species barriers. However, single-gene modifications often fail to provide lasting resistance against rapidly evolving pathogens. Consequently, stacking multiple genes with integrated defense mechanisms—also known as gene pyramiding—has emerged as a promising strategy for achieving more stable and broad-spectrum resistance (Gentzel et al., 2022; Dormatey et al., 2020). The gene pyramiding technique is one of the modern Techniques. Methods for producing new strains in modern molecular breeding programs involve combining several genes with a known effect into one genetic model (Werkissa, 2024). This is very important for plant breeders, especially resistance to biotic stresses. What distinguishes this resistance is its ability to endure, as overcoming the resistance of the host plant requires more mutations in the pathogen. In addition, this technique allows for long-term tolerance to the factors causing biotic and abiotic stresses in crops by giving resistance to different isolates and strains using a wide range of resistance genes (Joshi and Nayak, 2010).With recent developments in breeding science, particularly in recent years, new molecular techniques have emerged, enabling us to breed crops more precisely and in less time (Su et al., 2019). Breeding and variety development processes have relied on traditional breeding methods (Bhat et al., 2021). Using backcrossing, gene accumulation, and repeated selection to introduce new traits, gene pyramiding based on selection with molecular markers is crucial in breeding processes (Gupta et al., 2010). This technique reduces the number of generations that the breeder must analyze through molecular profiling of the stacked genes. With the availability of numerous molecular markers in addition to genetic maps, breeding has become much easier (Lema, 2018). Plant pathogen resistance is one of the few examples that can benefit from molecular markers (Dormatey et al., 2020). Gene pyramiding is a key application of molecular markers in plant breeding, particularly when identifying genotypes containing the desired genes for pyramiding (Chukwu et al., 2019). This process involves selecting one or more genes simultaneously, which are then used to enhance plant resistance. This is known as molecular-assisted gene pyramiding (MAGP), a technique that saves time and effort compared to traditional breeding methods. A successful example of this technique is the pyramiding of multiple genes, including those associated with QTLs, quantitative trait loci from different parents, into a single genotype within a short period (Collard and Mackill, 2008). Accumulating multiple genes, including those associated with QTLs, into a single genotype ensures long-term and sustainable resistance (Nelson et al., 2018). A successful example of this technique in chickpea is the work of Kaur et al. (2022), who introduced the cry1Ac gene using Marker-Assisted Backcross Breeding into chickpea plants to resist the chickpea pod borer. It was also addressed by Bharadwaj et al. (2022), who developed a high-yielding chickpea variety resistant to Fusarium wilt using gene pyramiding through Marker-Assisted Backcrossing. It is considered one of the important strategies for developing and improving varieties in various crops by using a smaller number of generations compared to traditional breeding.

Another example of quantitative trait gene accumulation in wheat plants is the use of three QTLs, which increased resistance to Fusarium crown rot compared to the use of a single QTL (Zhang et al., 2013). Researchers have also worked on accumulating eight quantitative trait genes, resulting in increased yield and plant resistance (Tyagi et al., 2014). An impactful example of gene pyramiding with quantitative trait loci (QTLs) to boost maize productivity is presented by Wang et al. (2025), who developed 320 recombinant inbred lines (RILs). Their study identified 79 QTLs, including nine major ones, that showed additive effects on traits such as ear row count and ear length. Notably, strains with one to six major QTLs demonstrated a strong positive correlation between phenotypic values and the number of favorable haplotypes of major QTLs (FHMQs). This indicates that plants with multiple favorable haplotypes achieved higher productivity. Additionally, hybrids from RILs crossed with three test strains, which had parents containing three to four FHMQs, significantly outperformed those with parents possessing two or fewer FHMQs. These findings highlight the potential of strategic breeding to enhance maize yields.

As molecular markers play an effective role in stacking genes by stimulating resistance genes and reducing the use of chemical pesticides, they thus reduce their accumulation in food (Han et al., 2024). The use of gene combinations provides a sophisticated approach to increasing the genetic basis of resistance, and its compatibility with diverse management strategies further enhances its effectiveness (Tiruvaipati et al., 2022). Among the various defense-related genes, chitinases are pathogenesis-related (PR) proteins that hydrolyze chitin, a major component of fungal cell walls, thereby inhibiting spore germination and hyphal growth (Chet and Inbar, 1994; Haran et al., 1996). Similarly, stilbene synthase (vst-1) genes encode enzymes responsible for producing phytoalexins such as resveratrol, which enhance antifungal defense (Jeandet et al., 2021; Tian and Liu, 2020) In a 2018 study on several Iranian-origin commercial cotton varieties(Varamin, Khordad, Sahel, and Bakhtegan), the chitinase gene for resistance to verticillium wilt and the cry1Ab gene for resistance to cotton worm were used, using conventional crossbreeding of strains containing both genes with Iranian commercial varieties (Mirzaei et al., 2018). Stacking such genes has successfully improved resistance in several crops, including rice, wheat, and potato (Ghislain et al., 2019; Luo et al., 2021; Pradhan et al., 2015), but has rarely been tested in chickpea. The most common method for stacking multiple genes in a single plant is crossing between genetically modified plants with one or more resistance genes (Zhu et al., 2015). Although the gene stacking approach to create multiple resistances is not novel, no reports have described stacking chitinase and vst1 genes for resistance to Aschochyta blight (Aschchyta rabiei) in chickpea plants. The purpose of this study was to stack VST1 and Chitinase genes in chickpea lines carrying these genes by the sexual crossing method, then evaluate resistance to A. rabiei, confirm gene inheritance by PCR, and assess resistance using spore germination inhibition, detached-leaf, and whole-plant assays. The results provide new insights into the potential of gene stacking to improve chickpea resilience against Ascochyta blight and improve durable disease resistance.

## Material and methods

This study was conducted at the Faculty of Agriculture, University of Aleppo, Syria.

### Plant material

ICC 12004: *Cicer arietinum* L. (Desi chickpea) is a genotype derived from the germplasm collection of the International Crops Research Institute for the Semi-Arid Tropics (ICRISAT). This genotype serves as a standard international differential line used to identify various pathotypes of the pathogen responsible for chickpea diseases. It is particularly valuable in breeding programs aimed at developing disease-resistant chickpea varieties that are resistant to pathotypes 1, 2, and 3. However, it is susceptible to a newly identified, highly virulent pathotype 4 (Bayaa et al., 2004).

Genetically modified chickpea seeds of the N-292/T10 strain (modified from ICC12004, desi type) were used, which were altered at the International Center for Research in Dry Areas (ICARDA) by Khatib (2008) using *Agrobacterium tumefaciens* bacteria.

**Figure 1:**
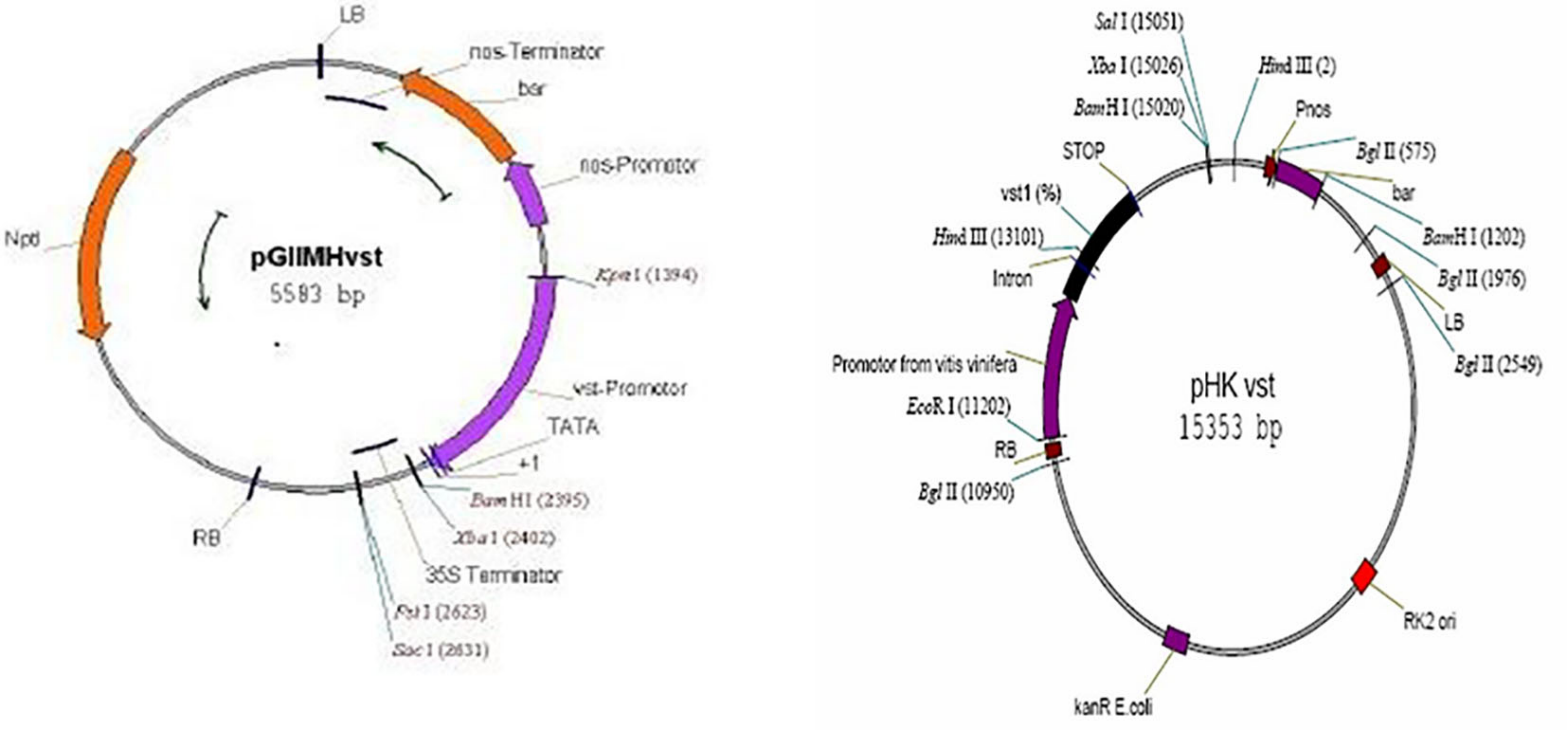
Plasmids utilized in the development of transgenic genes: pGII-vst for chitinase gene (Hassan, 2006 pHKvst-1 for vst1 gene (Kiesecker, 2000).

-The eighth generation of genetically modified chickpea seeds from strain/T8 N-346 (desi pattern) modified using *Agrobacterium tumefaciens* (Khatib and Baum, unpublished results) (**Figure 1**) as a vector to transfer the Chit30 chitinase gene from Streptomyces olivaceoviridis ATCC 11238 to increase its resistance to fungal pathogens.

- Fifth-generation genetically modified chickpea seeds from the/T5 N-52 (desi type) modified using *Agrobacterium tumefaciens* (Khatib and Baum, unpublished results) (**Figure 1**) as a vector to transfer the vitis stilbene (vst-1) gene from grapes to increase resistance to fungal pathogens. The seeds were sown at 15-day intervals under greenhouse conditions at a temperature of 22-25°C and a light period of 14 hours until they reached the flowering stage. Then, the crossing process was carried out during different time periods after confirming the presence of genes in the parents using the polymerase chain reaction.

### Cross pollination

Lines N292 and N346 were used as donor parents, while line N52 was used as replicate parents. When the plants reached the flowering stage, manual pollination was performed after the stigmas and anthers had matured. The pollination process was completed, and the pollinated flowers were isolated in hybrid bags. The crossing date and the parents used in the crossing were recorded on the special label. The fertilized pods were maintained until full maturity, and the first-generation seeds, F1, were collected and replanted to obtain second-generation seeds, F2. After crossing, the flowers were treated with 100 ppm gibberellic acid for 7 days to reduce ovary abortion. The polymerase chain reaction was used to confirm the presence of the gene in each generation.

### Detection of gene stacking by PCR

The cetyltrimethylammonium bromide (CTAB) method was used to extract DNA from the leaves of genetically modified and non-transgenic plants according to Doyle and Doyle (1991). The PCR mixture was prepared in a final volume of 20 μl containing 2 μl of PCR buffer (10x), 2 μl of magnesium chloride (25 mM), 1 μl of dNTPs (10 mM), 1 μl of each forward and reverse primer (10 pM), 1 μl of DNA template (50–100 ng), and 0.2 μl (1 unit) of Taq DNA polymerase. The primers in **Table 1** were used to detect the vst1 gene. PCR was performed for 29 cycles as follows: for the chitinase gene: 1 cycle at 95°C for 3 min (for initial lysis), 29 cycles of reactions at 94°C for 60 s (for lysis), 60°C for 60 s for annealing, 72°C for 60 s (for extension), and 72°C for 10 min (for final extension). For the vst-1 gene: 1 cycle at 94°C for 5 min (for initial lysis), 35 cycles of reactions at 94°C for 90 s (for lysis), 60°C for 90 s for annealing, 72°C for 60 s (for extension), and 72°C for 7 min (for final extension). The reaction products were separated on 1.3% agarose gels in 1X buffer. Agarose gels were stained with Ethidium bromide and photographed using a gel documentation system. Polymerase chain reaction (PCR) was used to detect the transgenes in the parental lines and gene stacking in the F1 generation. Gene segregation in the F2 generation was used to calculate the segregation percentage using the chi-square test.

**Table 1:** Primer sequences and the length of amplified fragments in PCR.

Primer name	Primer sequences (5' to 3')	PCR product size (bp)
chit	F: GGTGACATCGTCCGCTACAC R: GGTGTTCCAGTACCACAGCG	555
*vst1*	F: TTATAAATAC CCAACACTCACACCC R: CTTCTTGATCATTGATTTGTCACCTG	664

### Functional assay

#### Fungal isolation

The fungus was isolated from infected chickpea leaves, which were surface sterilized with 0.5% sodium hypochlorite for two minutes, then washed several times with sterile distilled water. The infected leaves were dried on sterile filter paper and then placed on chickpea seed extract agar (CDA) (40 g chickpea seeds, 20 g dextrose, 18 g agar/liter) and incubated at 20°C for 10 days with a 16/8-hour light/dark cycle.

#### Total protein extraction

The two genes in the container plants were induced in addition to the control by pricking their leaves with a thin sterile needle, then 1 g of leaves was collected 3 days after pricking. Each sample was crushed separately in 3 mL of 01. M sodium acetate extraction solution in a heat-sterilized porcelain mortar, and the extract was incubated in a refrigerator at 4°C for 2 hours. The extract components were separated by centrifugation at 4°C and 14, 000 rpm for 30 minutes. The supernatant was collected and sterilized by filtration through 022. μm pore size filters.

#### Spore germination inhibition test

*Ascochyta rabiei* spores were harvested from fresh culture dishes in 10 ml of sterile distilled water in an isolation chamber by gently swirling them, then the liquid was withdrawn, and the spores were counted using a red blood cell counting slide and adjusted to 1 × 6 10 spores/ml. The experiment was carried out in 1.5 ml test tubes according to a completely randomized design (CRD) (3 treatments × 4 concentrations × 3 replicates = 36). Ten μl of the previous spore suspension was added to each test tube. The enzyme extract was diluted with liquid chickpea culture (CD) to a final volume of 100 μl and three replicates, according to the following ratios:

0– Liquid culture: 3 extracts (0:90) μl+ 10 μl spore suspension, 1 - Liquid culture: 2 extracts (60:30 μl + 10 μl of spore suspension, 1 - Liquid culture: 1 extract (45:45) +10 μl spore suspension, 0 - Liquid culture: 3extract (0:90) + 10 μl spore suspension. The tubes were incubated at 22°C for 3 days. The mixture was then diluted for each treatment/concentration at a ratio of 1:100, and 100 μl was spread on (chickpea dextrose agar). The plates were incubated under appropriate conditions (temperature 22°C, 16/8 light/dark) for 8 days. Readings were taken using a. The colonies formed on the plates for the different treatments and concentrations were counted. The results were statistically analyzed using GenStat 15 software for ANOVA analysis, calculating the least significant difference between means at a significance level of 5%.

### Detached leaves

Leaves were collected from plants containing the two genes and from control plants at a rate of five replicates per treatment. The leaves were cleaned with 70% alcohol for 30 seconds and then placed in 0.5% sodium hypochlorite for 2 minutes. The leaves were washed with sterile distilled water for one minute three times and then placed on sterile filter paper to dry. The leaves were then transferred to agar-water dishes, with one leaf placed in each dish and secured in place. 1× 105 suspension was prepared, and each leaf was treated with 5 μl of the suspension.°The dishes were covered with parafilm and placed in an incubator at a temperature of 22°C with a 16/8-hour light/dark cycle. The assignment was taken after 12 days to calculate the disease severity (DS) in each plate on a 0-10 scale (Harijati and Keane, 2012).

**Figure 2:**
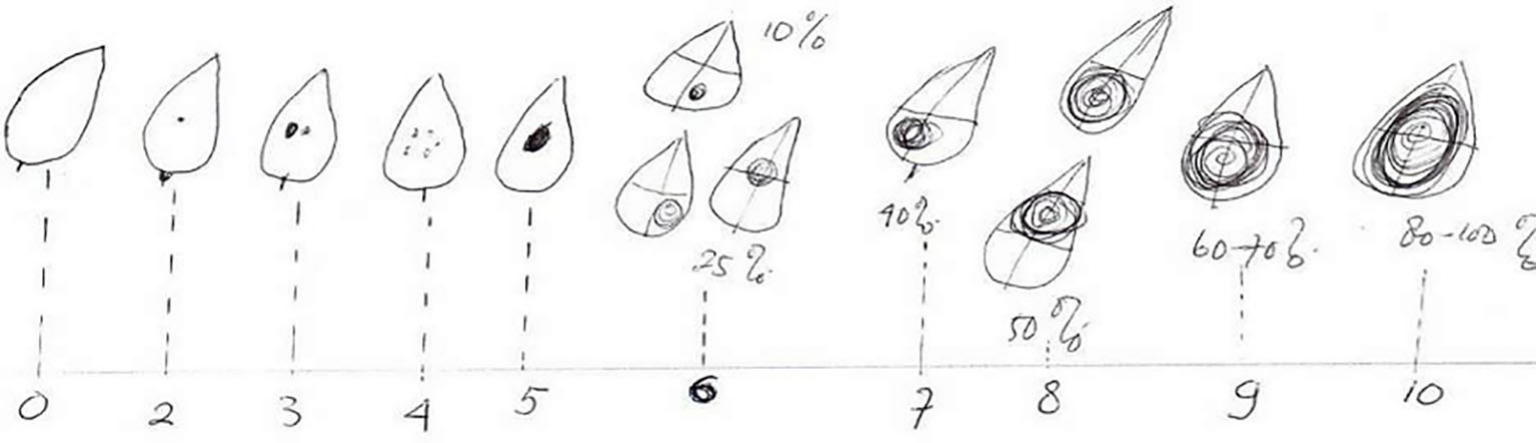
Diagram (Harijati and Keane, 2012) showing relative areas of necrotic and pycnidial lesions used to Assess disease severity (DS) on a leaflet on a scale of 0to 10.


Where a reading of 0 indicates no spot, and 2, 3, 4, and 5 indicate the presence of a single infected spot with an infected area of ​​approximately 10%, while 6, 7, 8, 9, and 10 indicate 25%, 30%, 40%, 50%, 80%, 60%, 70%, and 100%, respectively. The genotype was considered highly susceptible, moderately susceptible, moderately resistant, or highly resistant if the disease severity value was greater than 40, 25-39, 20-24, and 5-19, respectively. (**Figure 2**) The disease severity on a whole leaf was determined by using the following formula:

$$DS=\frac{\sum \left(\text{no}.\;\text{of}\;\text{leaflets}\;\text{in}\;\text{category}\times \text{category}\;\text{value}\right)}{\text{Total}\;\text{no}.\;\text{of}\;\text{leaflets}\times 10}\times 100$$

A chickpea genotype was regarded as highly susceptible, moderately susceptible, moderately resistant, or highly resistant if the disease severity value was >40, 25 - 39, 20 - 24, or 5 - 19, respectively. Disease incidence (DI) was calculated according to the following formula:


$$DI=\frac{\left(\text{no}.\;\text{leaflets}\;\text{with}\;\text{pycnidial}\;\text{lesions}+\text{no}.\;\text{leaflets}\;\text{with}\;\text{necrotic}\;\text{lesions}\right)}{\text{Total}\;\text{no}.\;\text{of}\;\text{leaflets}}\times 100$$

The genotype was classified as highly susceptible, moderately susceptible, moderately resistant, or highly resistant if the incidence rate was greater than 50%, 30-49%, 20-29%, or 5-19%, respectively.

### Whole plant

Chickpea seeds containing the two transferred genes were planted in small pots with the same number of unmodified control seeds in a sterile soil mixture consisting of soil and peat moss in a ratio of 3:1 under controlled conditions of 2 ± 22°°C and 14hours of light/10 hours of darkness. The 4-week-old plants were sprayed with a suspension of Ascochyta fungus at a concentration of 1 × 106 spores/ml, placed in incubators, and moistened during the first three days until infection and symptom development occurred. The percentage and severity of infection were recorded 21 days after infection according to a scale of 1-9 (Chen et al., 2004).

## Results

### Gene stacking by genetic crossing

Crossbreeding between strains was carried out using two parents from the same plant strain, each carrying a different gene, with different flowering times for the parents. The results of the first generation F1 crossbreeding between the two strains N292× N52 showed that 29 seeds contained both genes, and we also obtained 35 seeds when crossbreeding between the two strains N346× N52 (**Table 2**), where the resulting hybrid contained both genes. These hybrids were left to self-pollinate under greenhouse conditions, and the genes were isolated as follows in the hybrid N52×N292: only 15 plants out of 29 in the first generation contained both genes, while the genes were isolated in the second generation in the strain N346×N52, where only 9 plants contained both genes.

**Table 2:** Number of seeds produced by the chickpea cross.

Male plant	Number of Male seeds	Female plant	Number of Female seeds	Number of Seeds F1	Number of seeds F2
Vst-H(N292)/ Desi	6	Vst-1(N52)/ Desi	7	29	15
Vst-H(N346) / Desi	5	Vst-1(N52)/ Desi	6	35	9
Total	11		13	64	24

*n292, n346 line carry chit gene, n52 line carries vst1 gene.

### Confirmation of gene stacking by PCR

**Figure 3:**
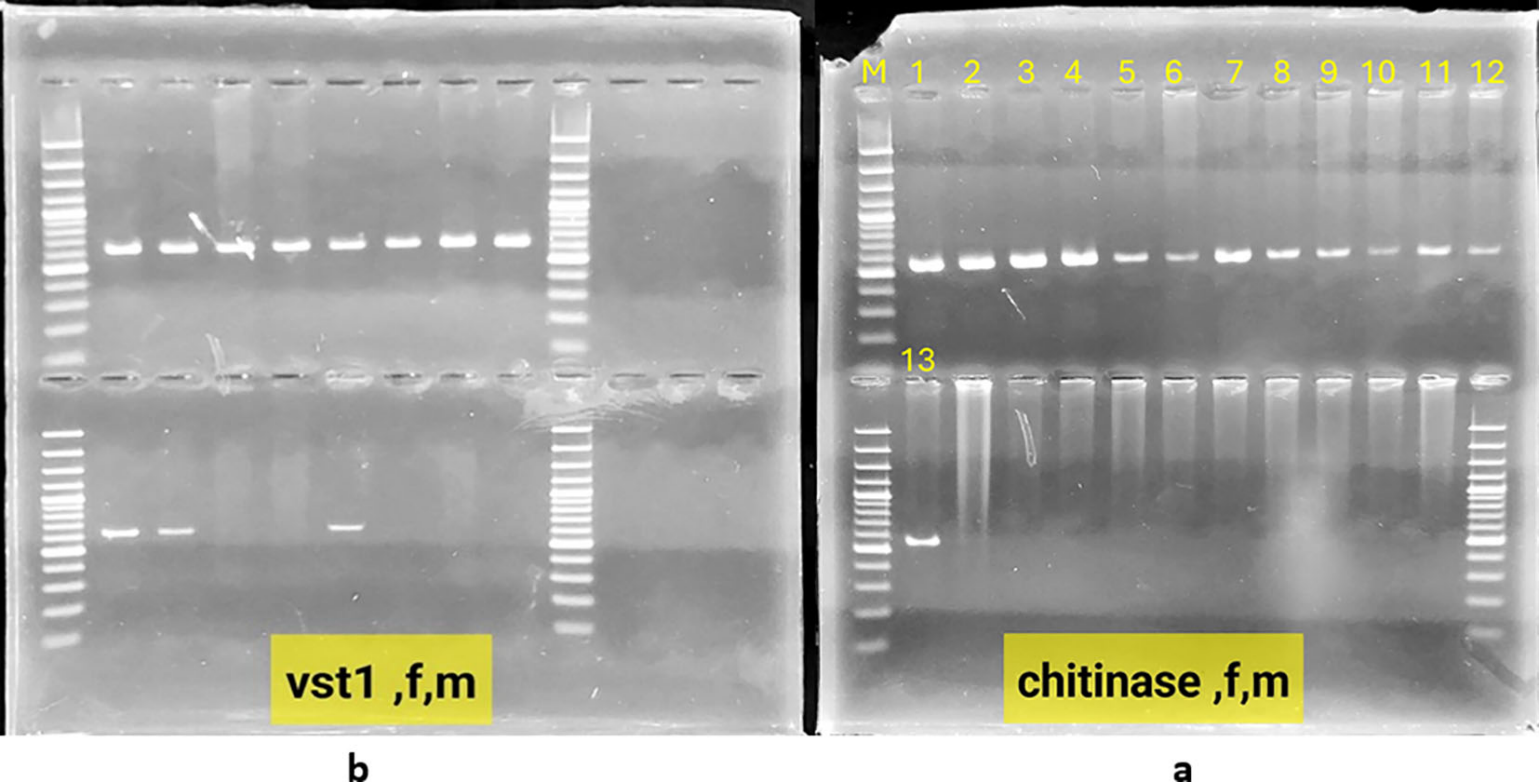
Electrophoresis on a 1.3% agarose gel of PCR products: **(a)** chit gene with amplification of 555bp in chickpea plants Lane M: molecular weight marker 100 bp DNA Ladder (Invitrogen BM211-01), lanes: 1-7: Chickpea plants genetically modified with the chitinase gene line n292, 8-13: Chickpea plants genetically modified with the chitinase gene line n346, **(b)** vst1 gene with amplification of 664bp Lane M: molecular weight marker 100 bp DNA Ladder (Invitrogen BM211-01, Lanes: 1-11: Chickpea plants genetically modified with the vst-1 gene line n52.

**Figure 4:**
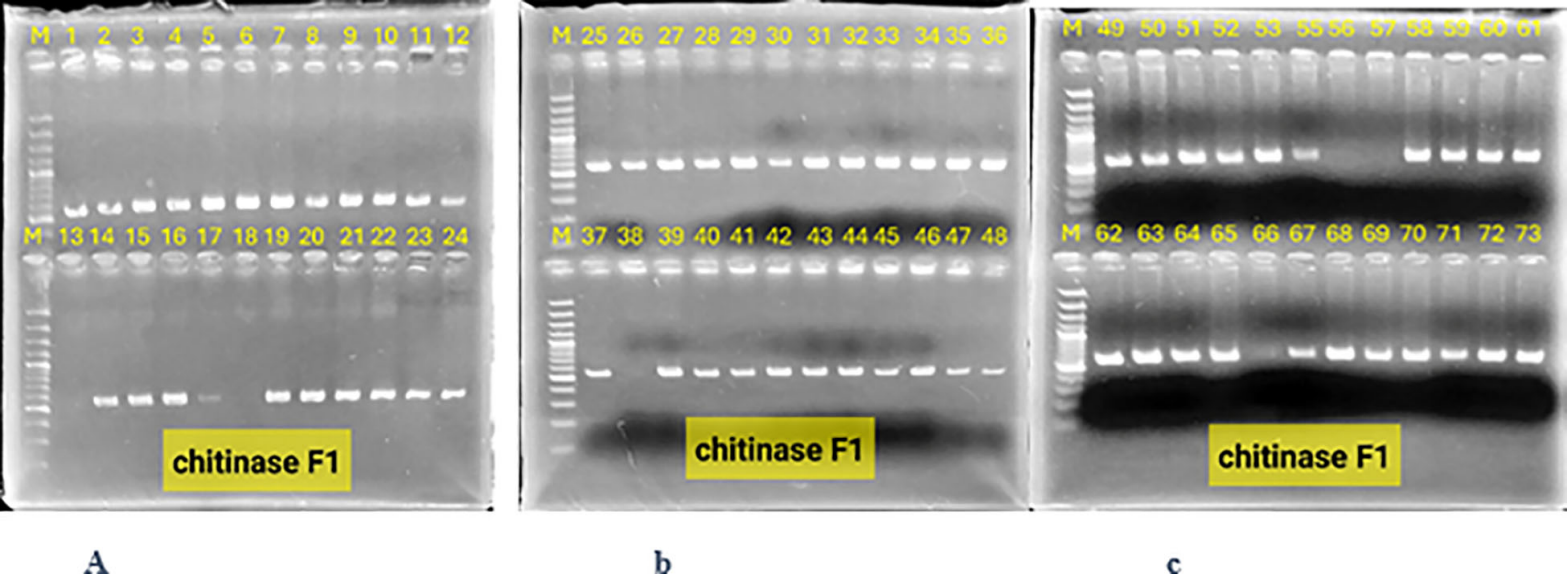
Electrophoresis on a 1.3% agarose gel of the products of the polymerase chain reaction for the chitinase, vst-1 genes in the first generation. **(A–C)** Lane M: molecular weight marker 100 bp DNA Ladder (Invitrogen BM211-01), lanes: 1-31: genetically modified chickpea plants with the chitinase gene line n292, 32-73: genetically modified chickpea plants with the chitinase gene line n346.

**Figure 5:**
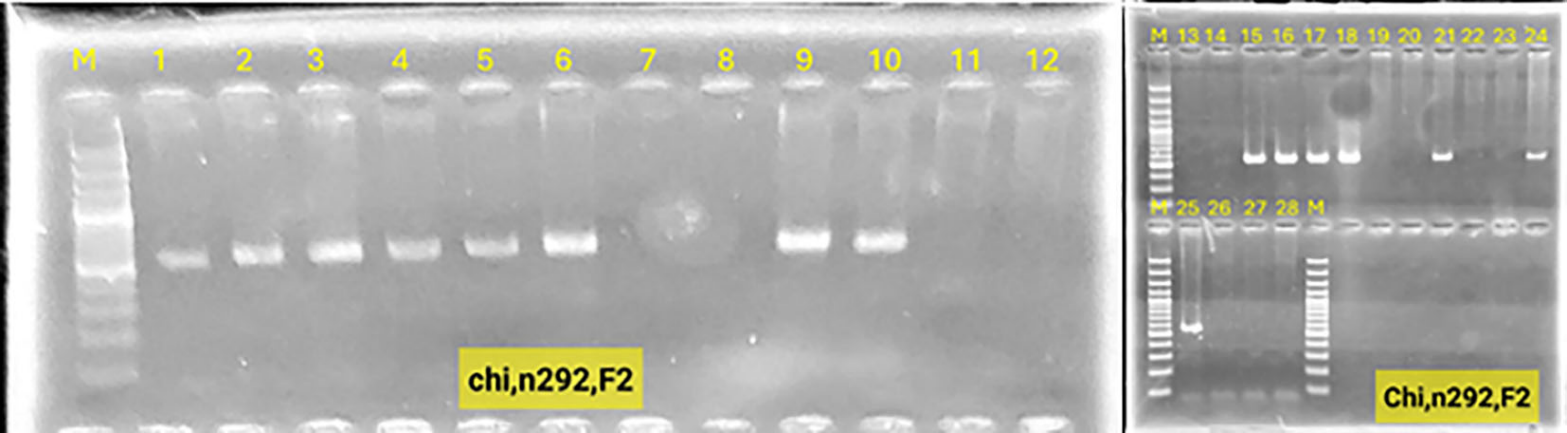
Electrophoresis on a 1.3% agarose gel of the products of the polymerase chain reaction for the segregation of the chitinase and vst-1 genes in chickpea plants Lane M: molecular weight marker 100 bp DNA Ladder (Invitrogen BM211-01), lanes: 1, 2, 3, 4, 5, 6, 9, 10, 15, 16, 17, 18, 21, 24, 25: representing gene stability in the second generation, while the absence of the gene in the remaining lanes is evidence of its segregation in hybrid n292 × n52.

**Figure 6:**
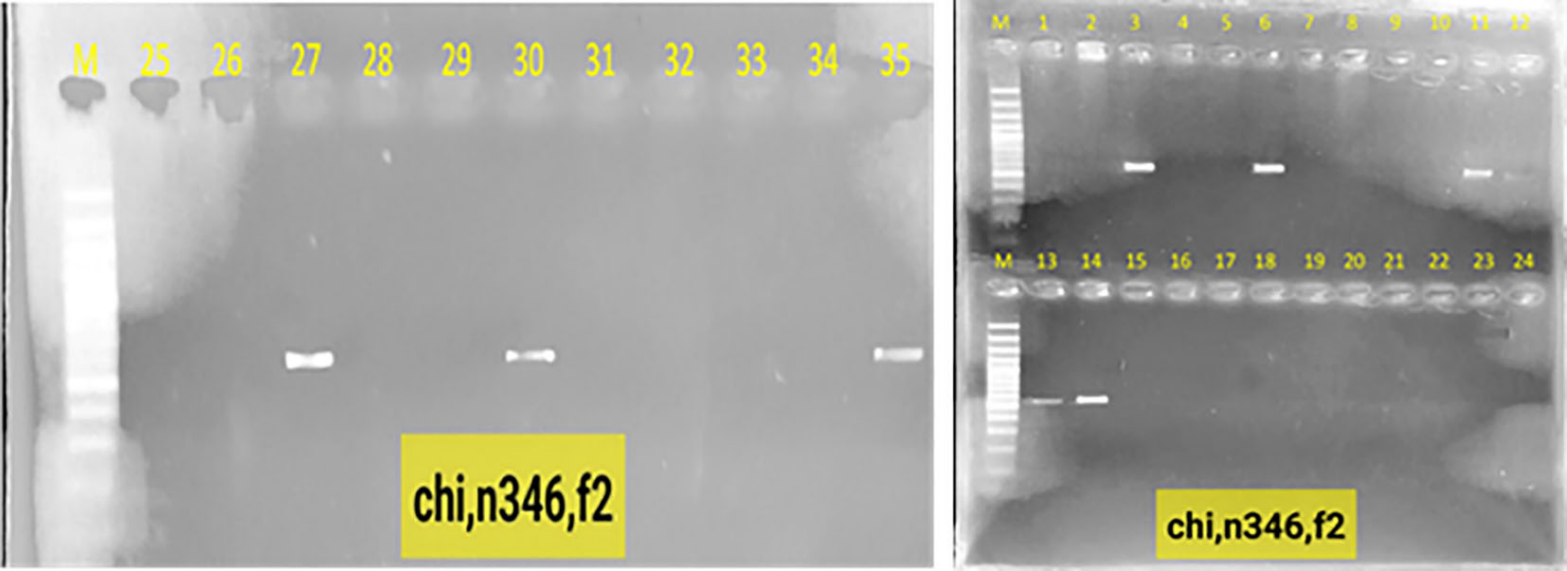
Electrophoresis on a 1.3% agarose gel of the products of the chain reaction for the segregation of the second-generation chitinase, vst-1 genes in chickpea plants Lane M: molecular weight marker 100 bp DNA Ladder (Invitrogen BM211-01), lanes: 5, 3, 6, 11, 12, 13, 14, 27, 30: represent the stability of the gene in the second generation, while the absence of the gene in the rest of the lanes is evidence of its segregation in hybrid n346× n52.

Polymerase chain reaction (PCR) was used to detect the presence of the vst1 and chi genes in the lines containing them, as well as to detect their transmission to subsequent generations. Stacking of vst1 and chi genes was performed successfully in F1 progeny. PCR analysis confirmed the presence of both genes, with expected fragment sizes of 555 bp (chitinase) and 664 bp (vst-1) (**Figure 3**). All F1 hybrids contained both genes, indicating stable inheritance from the parental lines (**Figure 4**). In the F2 generation, segregation of stacked genes was observed. For N292 × N52 hybrids, 15 out of 29 plants carried both genes, while for N346 × N52 hybrids, 9 out of 35 plants carried both genes. Chi-square analysis showed significant deviation from Mendelian expectations (χ² = 11.06 for N292 × N52; χ² = 19.45 for N346 × N52; P < 0.05), suggesting segregation distortion (**Figures 5**, **6**).

### Spore germination inhibition by protein extracts

**Figure 7:**
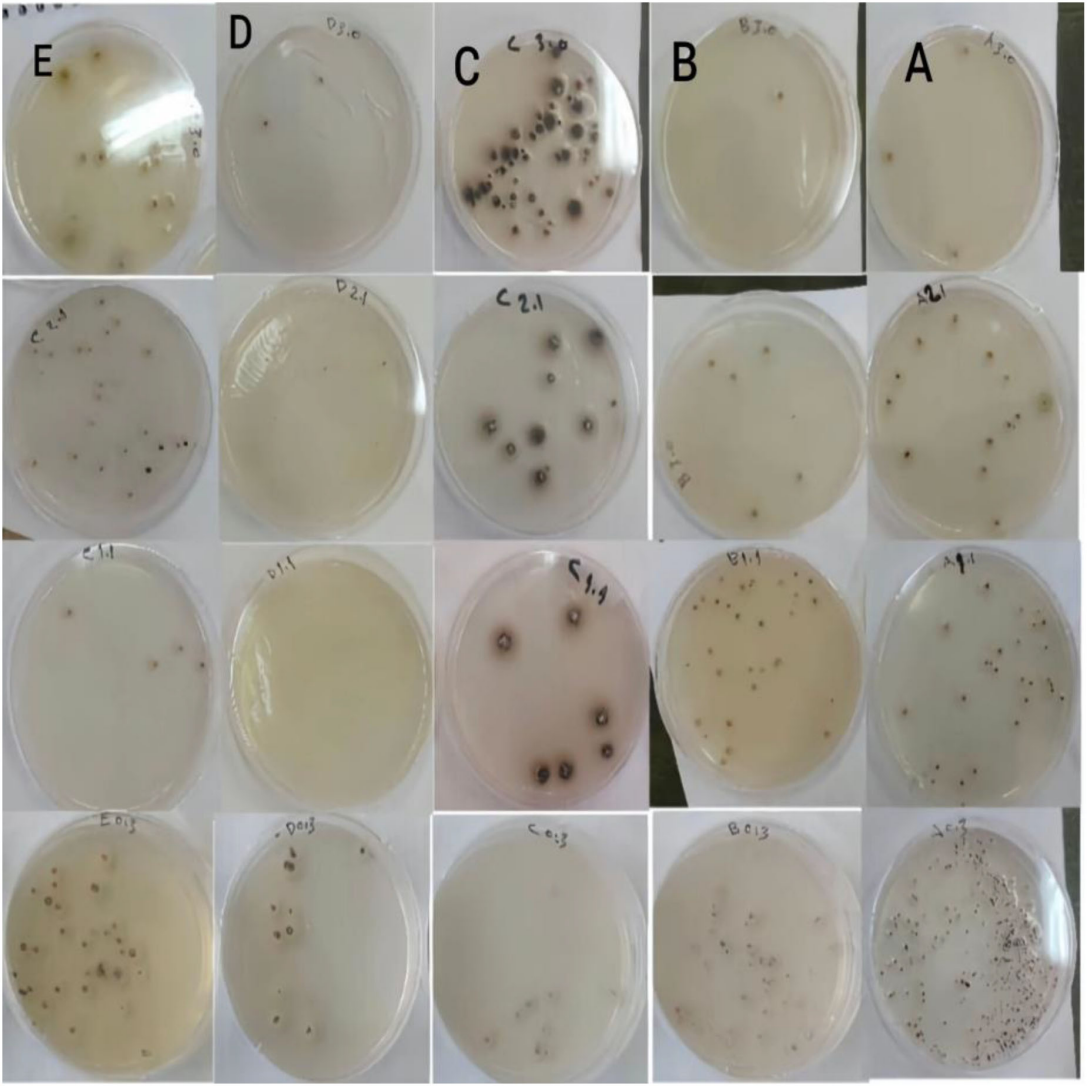
Fungal colonies formed at different dilutions for each treatment compared to the control **(A)** hybrid (N292*N52), **(B)** hybrid (N346*N52), **(C)** control (nontransgene), **(D)** line (N292), **(E)** line (N346).

Protein extracts from stacked lines significantly reduced spore germination compared to controls. At the highest extract concentration (3:0 ratio), spore germination was inhibited by 96% in N346 × N52 and 94% in N292 × N52, compared to only 12% in non-transgenic controls (**Table 3**, **Figure 7**). ANOVA confirmed highly significant treatment effects (F = 397.7, P < 0.001). Lower extract concentrations (2:1 and 1:1 ratios) also reduced colony formation, although less effectively. These results indicate that proteins from stacked plants exhibit strong antifungal activity.

**Table 3:** Number of Ascochytra fungus colonies produced after treating Ascochytra fungus spores with enzyme extract.

Treatment	Dilution	S1	S2	S3	Mean
A*	3: 0	6	3	3	4a
	2: 1	15	11	22	16bc
	1 :1	30	34	26	30de
	0:3	155	148	140	147j
B**	3: 0	1	6	1	2.6a
	2: 1	15	23	20	19.3bc
	1 :1	30	36	33	33de
	0:3	127	118	124	123h
C***	3: 0	26	33	30	29.6d
	2: 1	40	44	39	41f
	1 :1	44	55	58	52.3g
	0:3	160	144	156	153.3j
D****	3: 0	15	22	14	17bc
	2: 1	24	25	15	21.3c
	1 :1	33	25	36	31.3de
	0:3	150	144	146	146.6j
E*****	3: 0	9	14	13	12b
	2: 1	15	19	21	18.3bc
	1 :1	35	36	42	37.6ef
	0:3	136	133	132	133.6i
Lsd=7.71					

*A, hybrid (N292*N52); **B, hybrid (N346*N52); ***C, control (nontransgene);

****D, line (N292); *****E, line (N346); s1, s2, s3, replicate for each treatment.

### Detached-leaf assay

**Figure 8:**
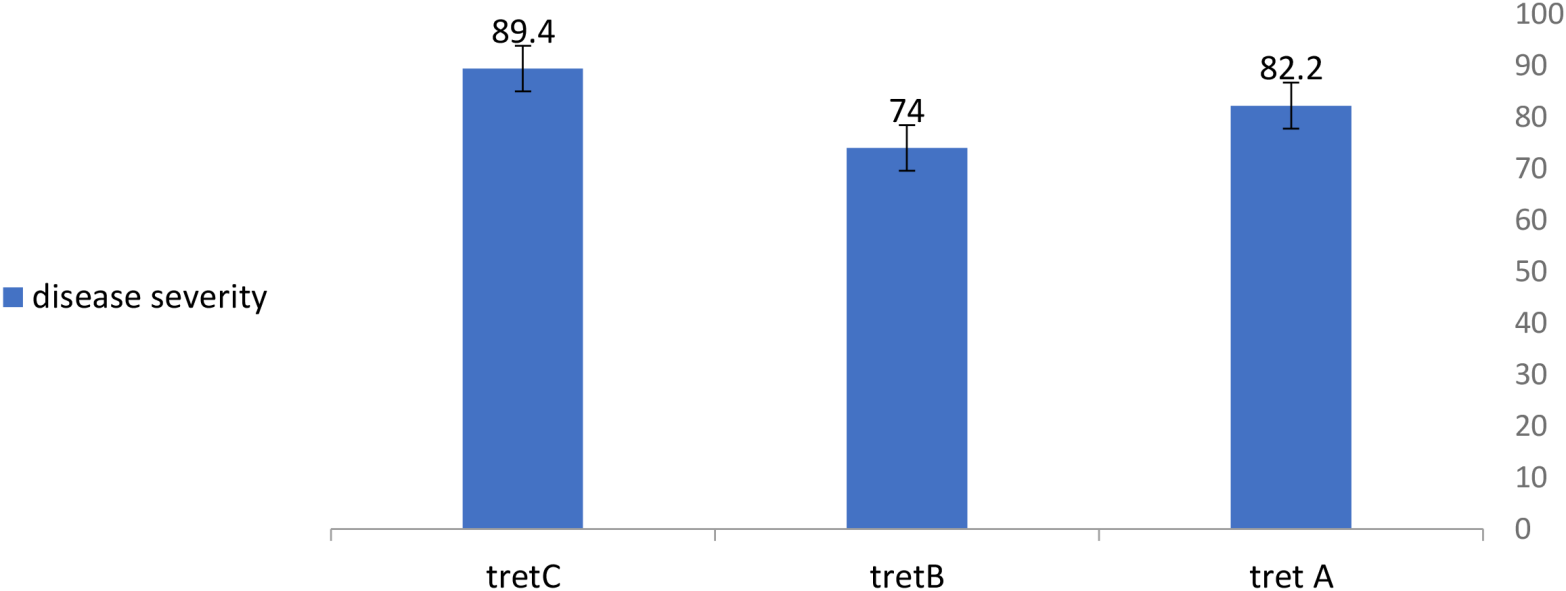
Disease severity of treatments with Ascochyta fungi using detached leaves.

All detached leaves developed *A. rabiei* lesions, but disease severity (DS) differed significantly between treatments (P = 0.003). Stacked hybrids showed reduced severity compared to controls (**Figure 8**). Mean DS values were:

N346 × N52: 74 (moderately susceptible) (**Table 4**).

**Table 4:** Average of disease severity with Ascochyta for each treatment using detached Leaves.

Treatment	D1	D2	D3	D4	D5	Mean
C	76	87	98	100	86	**89.4b**
B	68	71	69	82	80	**74a**
A	92	72	90	83	74	**82.2ab**
				13.63		**L.SD**

A, hybrid (N292*N52); B, hybrid (N346*N52); C, control (nontransgene); D1, D2, D3, D4, D5, replicate of disease severity for each plant.

N292 × N52: 82.2 (susceptible).

Control: 89.4 (highly susceptible).

**Figure 9:**
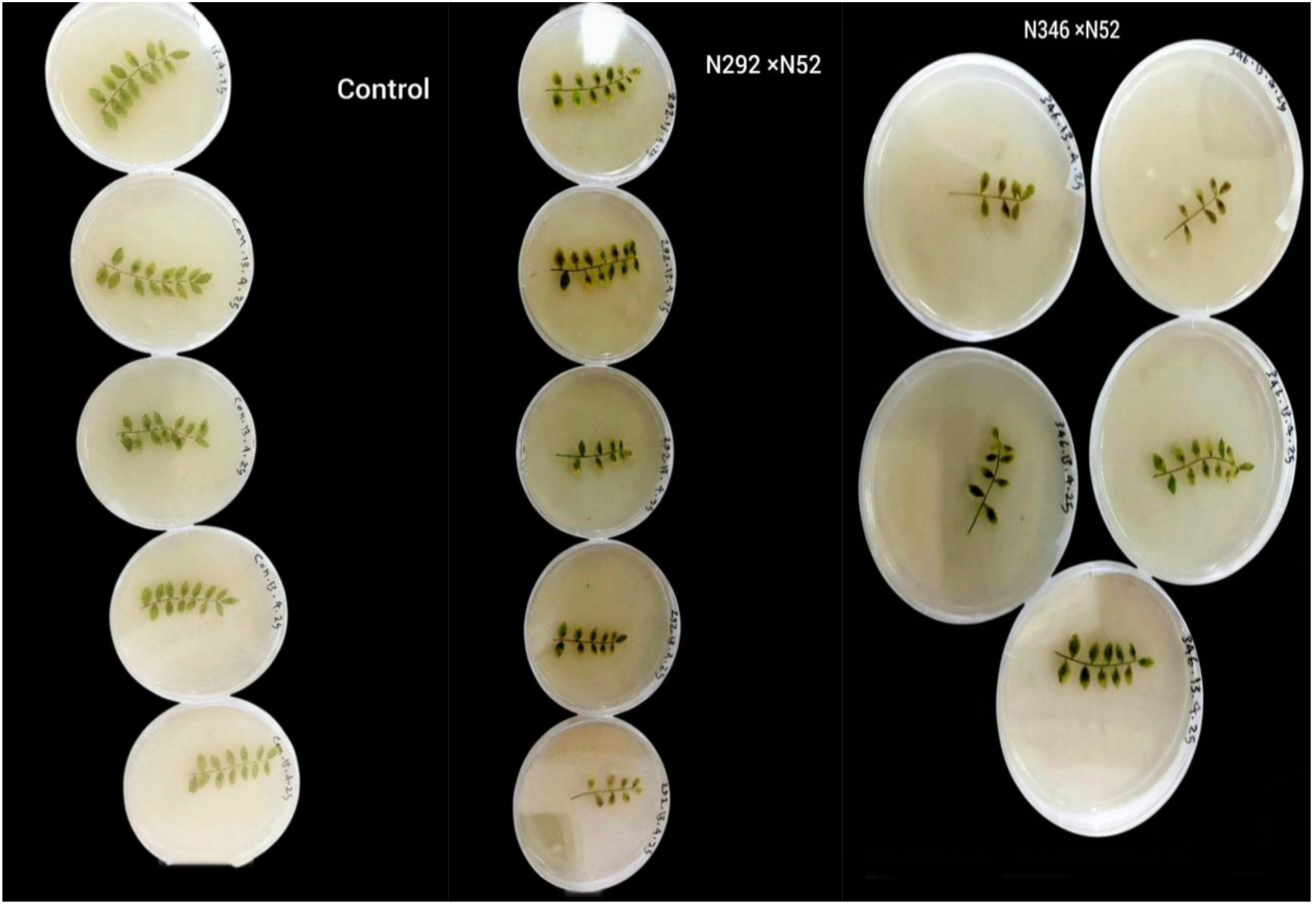
Variation in disease severity of Ascochyta fungus on genetically modified chickpea leaves compared to control leaves.

Although disease incidence (DI) exceeded 90% across treatments, stacked lines exhibited a clear reduction in lesion expansion, confirming partial resistance (**Figure 9**).

### Whole-plant assay

**Figure 10:**
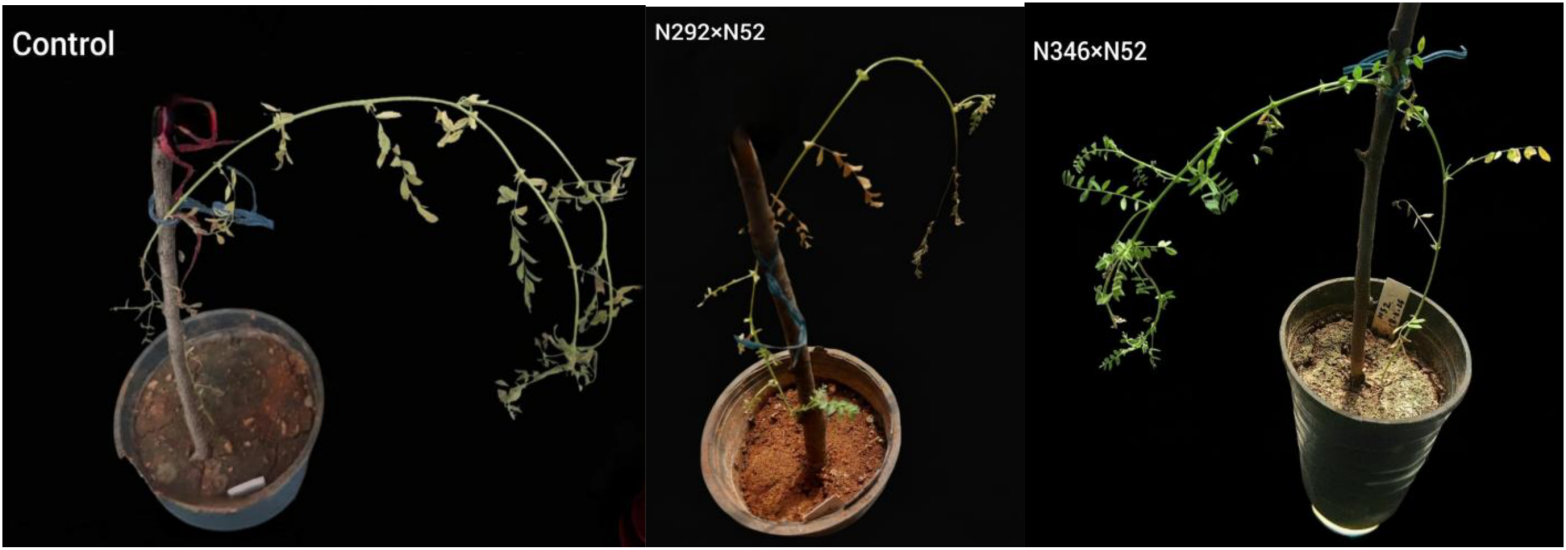
Variation in disease severity of fungus *A. rabiei* on genetically modified chickpea leaves (right) compared to control leaves (left).

In whole-plant inoculations, disease incidence was uniformly high (80–100%), indicating that gene stacking did not prevent infection. However, disease severity differed markedly among treatments. (**Figure 10**).

N346 × N52 plants showed moderate resistance (score = 4), with symptoms on ~15% of plants (**Table 5**).

**Table 5:** The scale for disease severity of Ascochyta blight infection proposed by Chen et al. (2004).

Description	Grade
The plant is healthy and immune, with no symptoms of disease.	1
High resistance: Small, indistinct spots appear on 3-5% of plants.	2
Resistant: Spots are easily visible on 6-10% of plants, no stem breakage, and the plant is completely green.	3
Moderate resistance: Large spots on leaves and stems, clearly visible on 11-15% of the plants	4
Tolerant: Symptoms appear on 16-25% of plants, and stem curling is observed on less than 10% of plants	5
Average susceptibility: Plant deterioration and wilting of growing tips; symptoms appear on 26-50% of plants	6
Moderate susceptibility: Symptoms appear on 51-75% of plants, stem lodging is observed on 50% of plants, with some plants dying, but at least three green leaves remain.	7
Susceptible: 76-100% of plants die, no green leaves, but the stem remains green, stem breakage, and infection on the pods.	8
High susceptibility: Death of the plant, no green parts on the plant.	9

N292 × N52 plants showed average susceptibility (score = 6), with ~50% of plants showing tip wilting.

Controls reached a severity score of 8 (susceptible), with extensive stem breakage and pod infection.

## Discussion

Ascochyta blight, caused by the fungal pathogen *Ascochyta rabiei*, is a major disease of chickpeas. A. *rabiei* infects all aerial parts of the plant and can cause severe yield loss (up to 100%) when conditions are conducive (Fanning et al., 2022; Pastor et al., 2022). The main reason for the emergence of A. *rabiei* strains is attributed to the accumulation of infectious inoculum (Ford et al., 2018), resulting from the failure to apply crop rotation or its weak implementation. Also, the genetic diversity of the fungal strains resulting from hybridization between the mating types of the fungus results in host resistance. Therefore, it is necessary to study the distribution and frequency of these patterns, which helps in predicting future pathogen patterns. Despite all this, the A. *rabiei* strains that reproduce asexually were able to overcome the resistance of their hosts (Sambasivam et al., 2020).

Rapid evolution of the pathogen threatens the future efficacy of both new sources of resistance and chemical control tools (Bar et al., 2021; Sharma and Ghosh, 2016). New tools that enable the development of improved approaches for the integrated management of A. *rabiei* are urgently required. Genetic engineering has proven successful in improving agricultural traits (Gentzel et al., 2022) by transferring one or more genes into the plant genome in a process known as gene stacking or gene pyramiding, This is credited with introducing multiple genes and traits into a single variety in what is known as gene stacking (Lundry, 2013), so that crops contain several genes to enhance their resistance to different types of pathogens. The use of this technology has also been shown to systematically increase resistance to pathogens, as it has increased the genetic basis for resistance in addition to its compatibility with management strategies, which greatly enhances its effectiveness (Tiruvaipati et al., 2022). To date, most genetic engineering research for disease resistance has relied primarily on identifying gene loci associated with quantitative traits (QTLs), along with understanding the mechanism and nature of infection and the genetic diversity of resistance genes in chickpea cultivars. Later, molecular markers (MAS) were used to link loci associated with quantitative traits to resistance to Ascochyta blight (Garg et al., 2018). However, it should be noted that there are no reports or research papers on combining multiple genes to resist this Ascochyta fungus to date. What distinguishes the gene combination technique is the increased resistance to pathogens compared to plants containing a single gene. Of interest in this study is the production of chitinase, a protein associated with the disease, vst1 stelbin, in response to microbial infection. Our study revealed that stacking chitinase and vst-1 in chickpea confers improved tolerance to Ascochyta *rabiei*, primarily by reducing disease severity rather than preventing infection. PCR confirmed stable inheritance of both genes in F1 hybrids, although segregation ratios deviated from Mendelian expectations in F2. Such segregation distortion has also been reported in other transgenic crosses and may reflect genetic background effects or reduced fitness of stacked genotypes (Dormatey et al., 2020). Protein extracts from stacked plants showed strong antifungal activity, inhibiting spore germination by more than 90%. This aligns with the known role of chitinases in degrading fungal cell walls (Chet and Inbar, 1994; Haran et al., 1996) and with reports of stilbene synthases (vst genes) producing phytoalexins such as resveratrol, which inhibit fungal growth (Jeandet et al., 2021). The combined activity of these two defense mechanisms likely explains the superior antifungal effect observed here. In detached-leaf and whole-plant assays, disease incidence remained high across all treatments, indicating that the stacked genes did not prevent infection. This result is consistent with what other researchers have reported, where they indicated that the stack of several genes may not provide effective resistance or may have a varying effect on disease tolerance and resistance. For example, the gluconase gene was unable to reduce the severity of infection by root fungi in alfalfa (Volpi et al., 2013). However, disease severity was significantly reduced, particularly in N346 × N52 hybrids, which were consistently more resistant than N292 × N52. This suggests that the stacked genes primarily slow disease progression rather than blocking pathogen entry. Similar patterns have been observed in potato varieties carrying stacked resistance genes, where infection occurred, but the lesion did not Expansion was suppressed (Ghislain et al., 2019). The difference between detached-leaf and whole-plant responses highlights the influence of physiological context. Detached leaves provide highly favorable conditions for fungal sporulation, often exaggerating susceptibility (Collard et al., 2001). Whole plants, however, showed greater resistance, suggesting systemic defense activation may improve the effectiveness of stacked genes. Our findings are consistent with previous reports that stacking complementary defense genes provides more durable resistance than single-gene approaches (Luo et al., 2021; Pradhan et al., 2015 Although genetically modified plants containing multiple genes for resistance to biotic and abiotic stresses have become essential for sustainable agriculture, reports of the stack of Ascochyta resistance genes in chickpea plants are, to date, few or none. We believe this research is the first to stockpile and evaluate the chitinase and vst1 genes against the most serious pathogen threatening chickpea cultivation in countries where this crop is grown. There are several studies on the accumulation of resistance genes. For example, the ChiC and Wasabi Defensin Genes (WD) were accumulated in genetically modified potatoes to resist both Alternaria solani and Fusarium oxysporum, where they showed broad resistance to these two fungi (Khan et al., 2014). It is also expected that the modified plants will show resistance to other major diseases. Similarly, in genetically modified tobacco, co-expression of chitinase and WD was obtained, which increased resistance to Fusarium wilt (Ntui et al., 2011). In another study conducted by Mehrotra et al. (2011, two types of cry genes, cry1Ab and cry1Ac, modified from Bacillus thuringiensis, were accumulated in genetically modified chickpeas to use them to control the chickpea pod borer, Helicoverpa armigera. Chitinase and antifungal proteins have been widely used in pathogen interactions. Their ability to degrade the fungal cell wall makes them an important factor in plant response (Chikara et al., 2012). A study conducted in Iran combined cry and chitinase genes to improve resistance to the cotton bollworm Heliothis armigera and Verticillium dahliae wilt in cotton plants. This was done by crossing lines containing the cry1Ab and chi genes with commercial Iranian cotton varieties. Insect and fungus *in vitro* bioassay showed resistance against cotton bollworm and Verticillium dahliae in plants containing the stacked transgenes (Mirzaei et al., 2018). Resistance genes to the following fungi: Trichoderma sp., Alternaria brassicae, A. brassicola, Verticillium longisporum, and L. maculans were tested under greenhouse conditions (Melander et al., 2006; Amian et al., 2011; Hassan et al., 2009; Richter et al., 2006).In a study on European pea cultivars, four fungal resistance genes—gluconase, chitinase, polygalacturonase, and stilbene synthase—were used to combat fungal pathogens in this plant. The results of the study showed that these genes exhibited tolerance to *Fusarium avenaceum*. They were tested individually or cross-bred under confined conditions for three years and compared with two German and three Canadian lines. However, they did not achieve the desired level of resistance and were unsuccessful in controlling the pathogen in the presence of an infectious inoculum (Kahlon et al., 2018). This is attributed to the fact that the co-expression of genes remains uncoordinated, even with physically linked genes (Maqbool and Christou, 1999), or that these genes are silenced before transcription. In this study, we analyze the effectiveness of combining the chit and vst1 genes into susceptible chickpea lines using conventional hybridization, which resulted in the stacking of these genes in the genome of the tested lines to improve their resistance to Ascochyta blight compared to non-transgenic plants. In addition, these new genetic resources resulting from this research contribute important material that can be used in chickpea resistance breeding programs. The development of genetically modified chickpea plants with multiple genes represents a useful line of defense against various invasive fungi and may lead to the emergence of varieties with broad and durable field resistance.

## Conclusion

The results of this study showed that gene stacking for resistance to Ascochyta *rabiei* by Traditional crossing between the studied lines enabled the stacking of the two genes in the resulting hybrids. This increases the plant’s immunity against the fungus that causes Ascochyta blight, meeting farmers’ needs. In addition to gene stacking, the segregation ratio in the second generation did not match the hypothetical values for Mendelian segregation ratios. By polymerase chain reaction, it was possible to detect the genes in the parents, stacked them in the first generation, and segregate them in the second generation, Furthermore, this study revealed that The number of viable spores of the fungus Ascochyta *rabiei*, decreased when treated with an enzyme extract containing the enzymes for the chitinase and vst-1 genes and Reduction in the amount of mycelium produced by spores or its failure to form after treatment with an enzyme extract from chickpea plants but The incidence of Ascochyta *rabiei* infection in chickpea leaves is not affected by the presence or absence of the two genes in the plant. Also, the decreased severity of infection by the fungus Ascochyta *rabiei* on chickpea leaves was tested using detached leaves due to the two genes. Therefore, these genes can be used and transferred to varieties sensitive to Ascochyta blight, for strategies in genetic improvement programs or the transition of traditional susceptible varieties, such as gab 1, gab2, to new resistant varieties with pyramided loci with major- and minor-effect resistance genes, thus hindering future resistance breakdowns by pathogens. The results of detached leave matched those of the whole plant evaluation in terms of infection rate and severity. It was the N346 hybrid that outperformed N52 in resistance to Ascochyta in the spore germination test, the detached leaf test, and the whole plant assay.

## Data Availability

The original contributions presented in the study are included in the article/supplementary material. Further inquiries can be directed to the corresponding author.
